# Utilization of amino acid for selective leaching of critical metals from spent hydrodesulfurization catalyst

**DOI:** 10.3389/fchem.2022.1011518

**Published:** 2022-10-10

**Authors:** Idol Phann, Yu Tanaka, Sae Yamamoto, Naoko Okibe

**Affiliations:** Department of Earth Resources Engineering, Kyushu University, Fukuoka, Japan

**Keywords:** spent catalyst, amino acid, alanine, critical metals, molybdenum, cobalt

## Abstract

While spent catalysts can cause serious environmental pollution, they can be considered an essential secondary metal source due to their high critical metal grades. The formation of the amino acid-metal complex is often seen in nature, and its potential application in hydrometallurgy can be foreseen. Alanine (Ala) was first screened as the most effective type of amino acid to be used for the selective leaching of spent hydrodesulfurization catalyst (consisting of MoS_2_ and Co_3_S_4_ supported on Al_2_O_3_, at 10% Mo and 2.4% Co grades). The sequential 3-step leaching (Step-1: Alkaline Ala leaching at 45°C, Step-2: Hot water leaching at 70°C, Step-3: Second alkaline Ala leaching at 45°C) was conducted where the role of Ala was found to be at least three-fold; 1) maintaining alkalinity by amino acid’s buffering capacity to assist Mo leaching, 2) selectively precipitating Co by forming Co-Ala complex with a distinctive pink color, which can readily re-dissolve in hot water to be separated from spent catalyst particles. 3) Effectively suppressing unwanted dissolution of Al throughout the reaction without needing pH control. Consequently, highly metal-selective, two separate Co-rich (<1% Mo and 79% Co dissolved, Al not detected) and Mo-rich (96% Mo, 19% Co, and 2.1% Al dissolved) leachates were obtained. This study highlighted the potential utility of amino acids as non-toxic, alternative metal lixiviant as well as a metal precipitant for selective leaching of critical metals from spent hydrodesulfurization catalyst.

## 1 Introduction

The demand for low-sulfur fuels is increasing due to the implementation of stricter environmental regulations in the last decade worldwide. Nonetheless, the processing of heavier crude oil with higher sulfur, nitrogen and metal contents is growing, necessitating the increased use of hydrodesulfurization catalysts in petroleum refineries (Marafi and Stanislaus, 2007; Akcil et al., 2015; Li et al., 2015). Hydrodesulfurization catalysts generally consist of the MoS_2_ active phase with Co or Ni promoters supported on γ-Al_2_O_3_ (Ihan, 2020). Deactivated catalysts (due to deposition of C, S and other heavy metals deriving from crude oil) are usually regenerated and reused multiple times before the end of the cycle. If not correctly handled, the spent catalysts can cause serious environmental pollution through the dissolution of toxic heavy metals and are thus classified as hazardous waste by the environmental protection agency in the United States (Ihan, 2020).

Despite such environmental concerns, spent catalysts are regarded as an essential secondary metal source due to their high metal grades (e.g., 4%–12% Mo, 1%–5% Ni, 0%–4% Co, 0%–0.5% V, 15%–30% Al; Park et al., 2006; Pinto and Soares, 2012, Ilhan, 2020). Hence, a number of studies attempted to recover valuable metals from spent catalysts through pyrometallurgical, hydrometallurgical and pyrohydrometallurgical routes (Zeng and Cheng, 2009). Pyrometallurgical processes are generally highly energy intensive, and the emission of harmful gases is a major disadvantage (Ihan, 2020). Although hydrometallurgical processes are considered less energy-consuming and the reactions more flexible and readily controllable, the use of concentrated acids, ammonia and other harmful chemicals can still cause secondary pollution and health and safety concerns.

Due to the toxic nature of such chemical lixiviant used in the conventional hydrometallurgy processes, researchers have been attempting to search for more environmentally benign, sustainable alternatives for the leaching of valuable metals from both natural ores and waste materials (Asghari et al., 2013; Astuti et al., 2016; Dewi et al., 2020). Organic acids such as citric acid, oxalic acid, gluconic acid, malic acid and succinic acid can be fermented by some filamentous fungi and/or bacteria out of renewable and waste materials as feedstock (Alonso et al., 2015). The mechanism of metal leaching with chemical or biogenic organic acids is based on the combination of the acid leaching reaction (Eq. 1) and metal-organic acid complexation (Eq. 2).

$$MeO+{2H}^{+}={Me}^{2+}+H_{2}O$$ (1)

$${Me}^{2+}+H_{2}-A=Me-A+{2H}^{+}$$ (2)

More recently, the utility of glycine (Gly), the simplest amino acid, was reported to be effective as a “green” metal lixiviant (Eksteen et al., 2017; O’Connor et al., 2018; Oraby et al., 2019; Li et al., 2020). In the alkali condition, Gly (plus oxidizing agent such as air/O_2_, H_2_O_2_, Cu^2+^) leached Cu, Au and Ag from their pure foils and oxide/sulfide minerals. Gly exists as H_2_NCH_2_COO^−^ (Gly^−^, glycinate anion), ^+^H_3_NCH_2_COO^−^ (H(Gly), zwitterion) or ^+^H_3_NCH_2_COOH (H_2_(Gly)^+^, glycinium cation) depending on the solution pH. Cuprous (Cu^+^) and cupric (Cu^2+^) ions were suggested to complex with both zwitterion and glycinate anion, with the latter being more stable (Li et al., 2020). Glycine solution was also shown to be effective in extracting base and precious metals from waste printed circuit boards by employing a two-stage process: Alkaline Gly solution was applied to leach Cu, Al, Pb, and Zn in the first stage, followed by the second stage for Au and Ag leaching in Gly solution in the starved cyanide environment (Oraby et al., 2019).

In hydrometallurgical reactions in general, the recovery of target metals from polymetallic leachates can become complicated, especially by the excessive solubilization of non-target metals (Hamza et al., 2018). In the case of spent hydroprocessing catalysts, the wide usage of porous media as the catalyst support leads to the unwanted dissolution of Al along with the target metals (Angelidis et al., 1995). Previous leaching studies on this particular waste stream utilized conventional inorganic acid or base (Angelidis et al., 1995; Barik et al., 2012; Pinto and Soares, 2012) as well as organic acids (Arslanoğlu and Yaraş, 2019). However, the low leaching selectivity remained to be solved. While use of EDTA for Ni recovery under the microwave condition led to a better selectivity (Pinto and Soares, 2013), its cost effectiveness needs to be improved especially for complex multi-metal compounds (Feng and van Deventer, 2011). The use of amino acids for this waste stream has not been reported yet. However, amino acids were shown to have little to no interaction with acid-consuming components such as Al and Fe, thus thought to be a good replacement for many other lixiviant in the leaching of natural minerals as well as waste printed circuit boards (Feng and van Deventer, 2011; Oraby and Eksteen, 2013; Oraby et al., 2019). The economic and environmental merits of using Gly-based leaching at the industrial scale were emphasized by Eksteen et al. (2017).

In nature, the formation of amino acid-metal complexes is often seen within biological systems as metals are essential cellular components. Many living organisms make extensive use of transition metals (e.g., Co, Fe, and Mn), which are involved in various biochemical functions (such as electron carrier, catalysis and structural roles) and are frequently associated with active sites of proteins and enzymes. Such properties of transition metals led to the development of medicinal inorganic chemistry to design new metal-based drugs (Sodhi and Paul, 2019). Han and Chi (2010) also synthesized a variety of amino acid (Ala, Asn, Gln, His, Ile, Lys, and Pro)-metal (Fe, Cu, and Zn) complexes and emphasized the importance of the study in the applied biochemistry field. All 20 common amino acids (except for the simplest amino acid Gly; R = H) contain a chiral α-carbon. Each amino acid processes unique characteristics deriving from the size, shape, solubility and ionization properties of its side chain. Consequently, diverse reactions can be expected between different amino acids and metals. Furthermore, several amino acids (e.g., glutamic acid, alanine, aspartic acid, serine, tryptophan, isoleucine; Okafor, 2007) can be produced *via* fermentation. By fermenting such amino acids from organic waste streams eventually, additional values can be added to the process being sustainable, low cost, and environmentally friendly. Despite the potential application of such naturally-occurring amino acid-metal complexes in the field of hydrometallurgy, studies on this topic are yet highly limited (except for the study of Gly as mentioned above).

In order to address the above issues, this study attempted to exploit the potential utility of amino acids as non-toxic, alternative metal lixiviant for selective leaching of spent hydrodesulfurization catalyst. To our knowledge, this is the first report utilizing amino acids for the selective leaching of critical metals (Mo and Co).

## 2 Materials and methods

### 2.1 Sample preparation and characterization of spent hydrodesulfurization catalyst

The Mo-Co/Al_2_O_3_ type hydrodesulfurization catalyst (PT Pertamina (Persero), Refinery Unit IV Cilacap, Indonesia) was used in this study. As-received spent catalyst sample was ground by a planetary ball mill (Pulverisette-6; Fritsch, Tokyo, Japan) and dry-sieved to obtain a particle size of 75–150 µm prior to the leaching tests. The particle size distribution analysis was conducted using a laser diffraction particle size analyzer (Horiba, Partica LA-950). The mineralogical composition was analyzed by X-ray diffraction (XRD; Ultima IV; Rigaku, Tokyo, Japan) using Cu K*α* radiation, 40 mV, 40 kV, and a scanning speed of 2°/min. To analyze the elemental composition, 0.5 g of ground spent catalyst was digested in 9 ml of reverse aqua regia solution (HCl: HNO_3_ = 1:2 v/v) in the microwave (Ethos Plus, Milestone) by heating at 1,000 W to achieve 230°C in 30 min, and keeping at 230°C for 15 min, then allowing to cool to room temperature. The acid digestion leachate after the microwave treatment was left to evaporate before diluting with deionized water for ICP-OES analysis. The acid digestion was done in triplicate set-ups. The elemental composition was also analyzed by X-ray fluorescence spectroscopy (XRF) (Rigaku, ZSX Primus II, Akishima, Japan) as a comparison. The spent catalyst surface was sputter-coated with Au using a magnetron sputter (MPS-1S; Vacuum Device Inc., Tokyo, Japan) and observed by scanning electron microscope (SEM; Hitachi SU1000 FlexSEM 1000II, Tokyo, Japan) at an accelerated voltage of 20 kV.

### 2.2 Comparison of different acids as metal lixiviant

Analytical-grade reagents were used as lixiviant. All tests were carried out in 300 ml Erlenmeyer flasks containing 100 ml of different lixiviant and ground spent catalysts at a pulp density of 10% (w/v). Several amino acids (L-alanine (Ala), L-glycine (Gly), L-phenylalanine (Phe), L-glutamine (Gln) and L-arginine (Arg) at 0.5 M, pH_initial_ 11 with NaOH) as well as 0.5 M H_2_SO_4_ and 0.5 M citric acid were compared as representative inorganic and organic acids, respectively. All tests were done in duplicated flasks, incubated shaken at 150 rpm and 45°C. Samples were regularly taken to measure pH and metal concentrations (Mo, Co, and Al) by ICP-OES (LOD: Mo 7.9 μg/L; Co 7 μg/L; Al 28 μg/L).

### 2.3 Three-step leaching of spent catalyst

#### 2.3.1 Step-1: Alkaline L-alanine leaching

Erlenmeyer flasks (500 ml) containing 200 ml of 0.5 M Ala plus 3% (w/v) spent catalyst were prepared (pH_initial_ 11 with NaOH). Ala-free control flasks (pH_initial_ 11) were also set up in parallel. All flasks were incubated and shaken at 150 rpm and 45°C. Liquid samples were taken periodically to monitor pH and metal concentrations (Mo, Co, and Al) by ICP-OES.

#### 2.3.2 Step-2: Hot water leaching

In Step-1, the dissolution of Co was found to be accompanied by its simultaneous precipitation (regardless of the presence of Ala). Therefore, Step-2 attempted the selective re-solubilization of Co. The Step-1 solid residues (deriving from either Ala system or Ala-free control) were individually collected by centrifugation (10,000 G for 10 min; Suprema 21, TOMY), washed with deionized water four to five times until the pH dropped to 9.0, filtered and finally freeze-dried (EYELA, FDU-1200, Tokyo, Japan) before being applied for Step-2. In Step-2, 500 ml Erlenmeyer flasks containing 150 ml of deionized water plus 3% (w/v) of the Step-1 residue were prepared and incubated, shaken at 150 rpm and 70°C for 48 h.

#### 2.3.3 Step-3: Second alkaline L-alanine leaching

The Step-2 solid residues (deriving from either Ala system or Ala-free control in Step-1) were individually collected by centrifugation (10,000 G for 10 min), washed with deionized water and freeze-dried for later analyses. Only the solid residue derived from the Ala system was further processed in Step-3. In Step-3, two types of Ala solutions were compared; 1) pregnant Ala solution reused from Step-1 (pH_initial_ re-adjusted to 11 with NaOH) or 2) fresh 0.5 M Ala solution (pH_initial_ 11). One-hundred milliliters of Ala solution (either 1 or 2) and 3% (w/v) of the Step-2 solid residue were transferred into 300 ml Erlenmeyer flasks and incubated, shaken at 150 rpm and 45°C for 96 h.

At the end of Step-1, 2, and 3, washed and freeze-dried solid residues were subjected to XRD and SEM analyses. The final Step-3 residues were subjected to complete acid digestion to confirm their elemental compositions as follows; 0.1 g of the residue was digested in 10 ml aqua regia (HCl: HNO_3_ = 2:1 v/v) in the microwave (heated at 1,000 W for 30 min to reach 210°C, kept at 210°C for 15 min, then allowed to cool to room temperature). The leachate was then filtered and diluted with deionized water for ICP-OES analysis. The three-step leaching tests and final acid digestion was done in duplicate.

### 2.4 Chemical synthesis of Co-Ala complex as reference compounds

During Step-1 (Section 2.3.1), apparent Co-precipitates were formed. In order to identify this precipitate, the following three separate tests were conducted to synthesize Co-Ala complexes as reference compounds;

(i) The ethanol reflux method (Alam et al., 2019; modified): Fifty milliliters of 0.4 M Ala (natural pH) and 40 ml of hot ethanol containing 250 mM Co^2+^ (added as CoCl_2_· 6H_2_O) were separately prepared. The former was added drop by drop into the latter under vigorous stirring, followed by pH adjustment to 7.5 with Na_2_CO_3_. The reaction mixture was refluxed (in a beaker closed with aluminum foil) at 80°C under vigorous stirring for 3 h. The resulting purple precipitate was recovered by filtration (0.45 µm).
(ii) 500 ml Erlenmeyer flasks containing 200 ml of 0.5 M Ala (pH_initial_ 11) plus 50 mM Co^2+^ (added as CoSO_4_· 7H_2_O) were incubated shaken at 45°C and 150 rpm. After 48 h, the resultant pink precipitate was recovered by filtration (0.45 µm).
(iii) 500 ml Erlenmeyer flasks containing 200 ml of 0.5 M Ala (pH_initial_ 11) plus 6% (w/v) ground spent catalyst were incubated and shaken at 45°C and 150 rpm. After 3 h-incubation (before the initiation of extensive Co-precipitation), spent catalyst particles were separated from the leachate by filtration (0.45 µm). The solid-free leachate was then transferred into a new 500 ml Erlenmeyer flask and further incubated, shaken at 45°C and 150 rpm. After 48 h, the resultant pink precipitate was recovered by filtration (0.45 µm).

The resultant three types of Co-precipitates were compared by XRD and SEM. For qualitative elemental composition analysis, elemental mapping was done using EDS software (Aztec EDS, Oxford Instruments, United Kingdom).

## 3 Results and discussion

### 3.1 Characterization of spent catalyst

**FIGURE 1:**
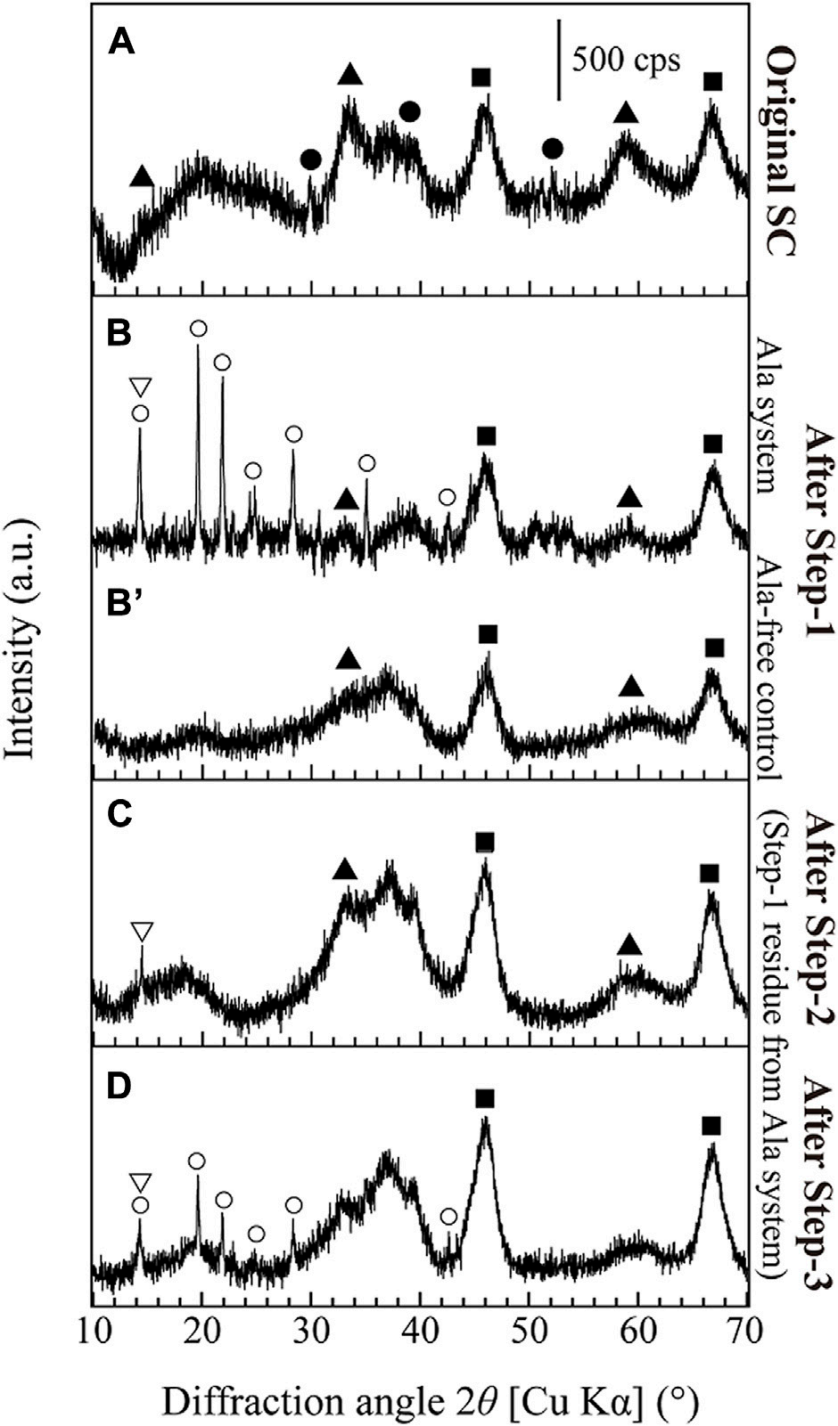
XRD patterns of; **(A)** the original spent catalyst, **(B,B′)** the leaching residue after Step-1 (comparison of with or without Ala, respectively), **(C)** the leaching residue after Step-2 and **(D)** the leaching residue after Step-3. Symbols: ▲ (MoS_2_; JCPDS No. 37-1492), ● (Co_3_S_4_; JCPDS No. 42-1448), ■ (Al_2_O_3_; JCPDS No. 75-0921), ▽ (AlO(OH); PDF No. 01-073-9093), ○ (Co-Ala complex; Figure 5).

As-received spent catalyst sample was quadlobe in shape with particle length varying from 1.5 to 5 mm (Figure 4A). The elemental compositions of the spent catalyst analyzed by XRF and acid digestion (followed by ICP-OES measurement) are compared in Table 1: According to the latter, critical metals such as Mo and Co were present at approximately 10% and 2.4%, respectively. The average particle size (P_50_) of the ground spent catalyst was 38.3 ± 1 μm. The XRD analysis confirmed that the spent catalyst comprises MoS_2_ and Co_3_S_4_ supported on Al_2_O_3_ (Figure 1A). Other contaminant metals such as As, Fe, S, and Ni (Table 1) were likely derived from the crude oil through the desulfurization process, leading to catalyst deactivation (Angelidis et al., 1995; Park et al., 2006; Ilhan, 2020). Similar values were also obtained from the XRF analysis (Table 1).

**TABLE 1:** Elemental composition of the as-received spent catalyst determined by XRF or acid digestion followed by ICP-OES analysis.

Element	XRF (wt. %)	Acid digestion followed by ICP-OES (wt. %)
Al	26.8	24.8 ± 0.27
Mo	13.1	10.1 ± 0.80
Co	3.4	2.4 ± 0.08
As	0.3	0.3 ± 0.02
Fe	0.2	0.2 ± 0.01
S	10.4	9.3 ± 1.13
Ni	0.1	0.04 ± 0

### 3.2 Effect of different acids on the spent catalyst leaching

**FIGURE 2:**
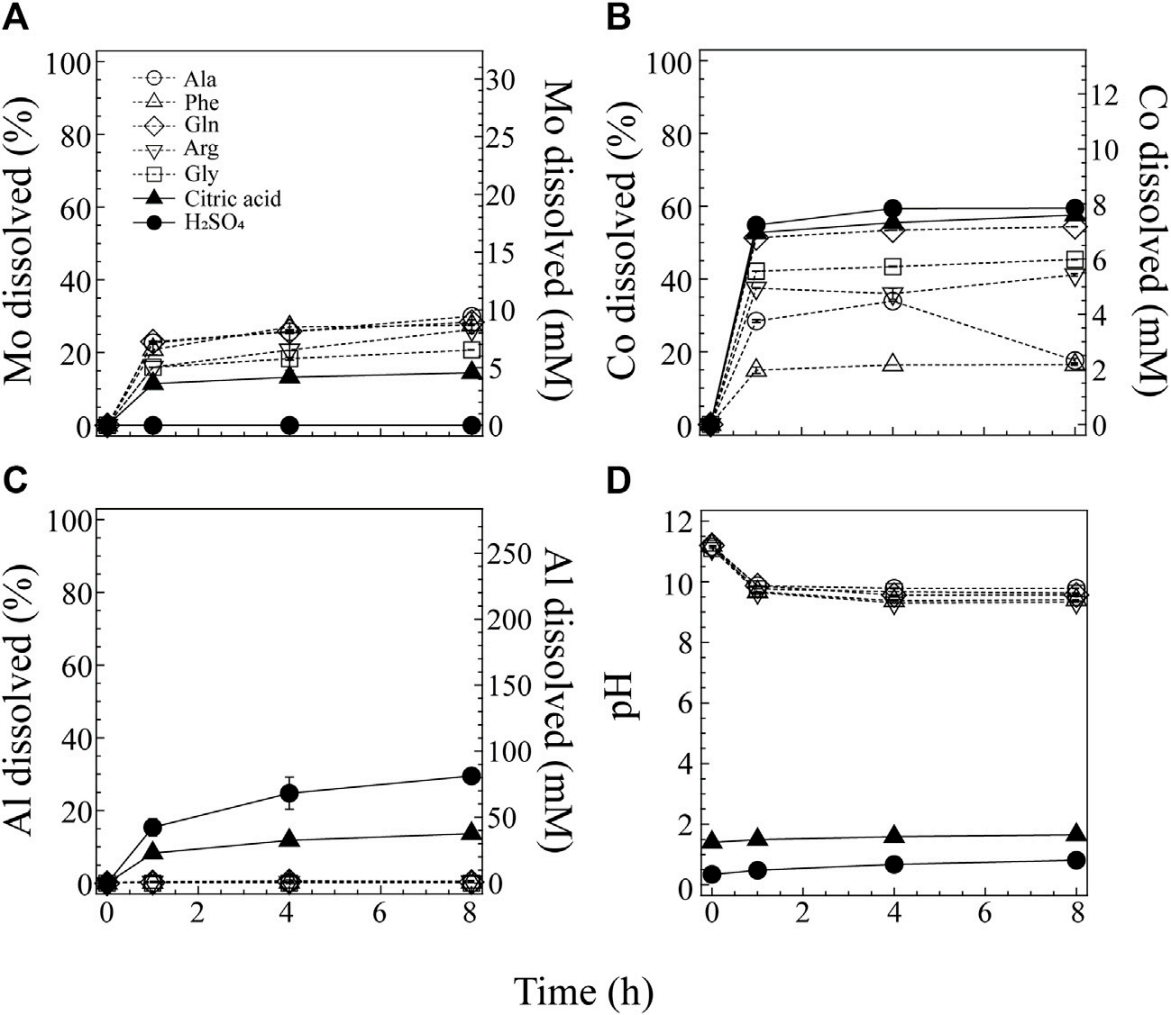
Comparison of 0.5 M sulfuric acid (●), 0.5 M citric acid (▲) and a variety of alkaline-amino acids (0.5 M; Ala ○, Phe △, Gln ◇, Arg ▽, Gly □) as a lixiviant for spent catalyst. Changes in the Mo dissolution **(A)**, Co dissolution **(B)**, Al dissolution **(C)** and pH **(D)** are shown.

Figure 2 compares different lixiviant: 0.5 M H_2_SO_4_, 0.5 M citric acid and several amino acids (0.5 M Ala, Gly, Phe, Gln or Arg; pH_ini_ 11). The use of H_2_SO_4_ as a conventional inorganic acid lixiviant was ineffective in the Mo leaching, while the dissolution of Co (70% at 48 h; Figure 2B) and Al (48%; Figure 2C) was the greatest of all. Citric acid showed the second lowest pH range (1.4∼1.9) after H_2_SO_4_ (pH 0.3∼1.8; Figure 2D), where the Mo dissolution was improved to ∼19% and Co and Al leached at 64% and 20%, respectively (Figures 2A–C). In contrast to these two acidic lixiviants, alkaline amino acids suppressed the Al dissolution to <1% (7.5 mM; Figure 2C). The pH trend was similar with all amino acids; i.e., pH_ini_ 11 quickly dropped to ∼9.5 but stabilized (Figure 2D). However, the metal dissolution behavior varied between the different amino acids. Gln showed the greatest dissolution of both Mo (38%) and Co (61%). Ala and Phe also leached an equivalent amount of Mo (∼34%) but seemingly dissolved and precipitated Co simultaneously. Gly and Arg leached a lesser amount of Mo than other amino acids (29% and 22%, respectively) and also a lesser amount of Co (49% and 39%, respectively) than Gln (Figures 2A,B).

Based on the pourbaix diagram for Mo (Supplementary Figure S1; Lyon, 2010), Co (Supplementary Figure S2; Chivot et al., 2008), and Al (Supplementary Figure S3; Pourbaix, 1974; Sukiman et al., 2012), Mo tends to ionize at neutral to alkaline pH. The use of H_2_SO_4_ was therefore ineffective for the acidolysis of Mo. However, despite its acidity (pH ∼1.8), citric acid solubilized Mo to some extent (Figure 2D), suggesting the possible involvement of complexolysis. When amino acids were used under the alkaline condition (pH_ini_ 11), the Mo dissolution likely depended on alkalosis according to the pourbaix diagram (Supplementary Figure S1). Still, the Mo dissolution trend was not identical between different amino acids, suggesting that there may be an additional effect caused by certain types of amino acids other than alkalosis, possibly complexolysis.

On the other hand, Co^2+^ is soluble in the wide pH range from strongly acidic to neutral pHs, while at alkaline pH Co tends to precipitate as Co hydroxides, according to the pourbaix diagram (Supplementary Figure S2). Despite the nearly identical pH value maintained throughout the leaching period (pH ∼10; Figure 2D), the Co dissolution profile varied largely between different amino acids. Some amino acids (such as Gln) seemed to even aid the dissolution of Co at this high pH, like other acidic lixiviants (Figure 2B). In contrast, Co was dissolved but precipitated simultaneously in the presence of Ala and Phe (Figure 2B). The dissolution trend of Al was highly pH dependent: Al dissolution was mostly suppressed under the alkaline amino acid conditions while H_2_SO_4_ greatly leached Al to 48% (Figure 2C), as expected from the pourbaix diagram (Supplementary Figure S3).

The contrasting leaching trend between Mo and Co was most evident when Ala and Phe were used, while unwanted Al leaching was effectively prevented (Figures 2A–C). Therefore, the following tests employed Ala as a simpler representative to attempt selective leaching of the two critical metals.

### 3.3 Selective leaching of Mo and Co in the alkaline L-alanine system

#### 3.3.1 Sequential 3-step leaching

##### 3.3.1.1 Step-1: Alkaline L-alanine leaching

**FIGURE 3:**
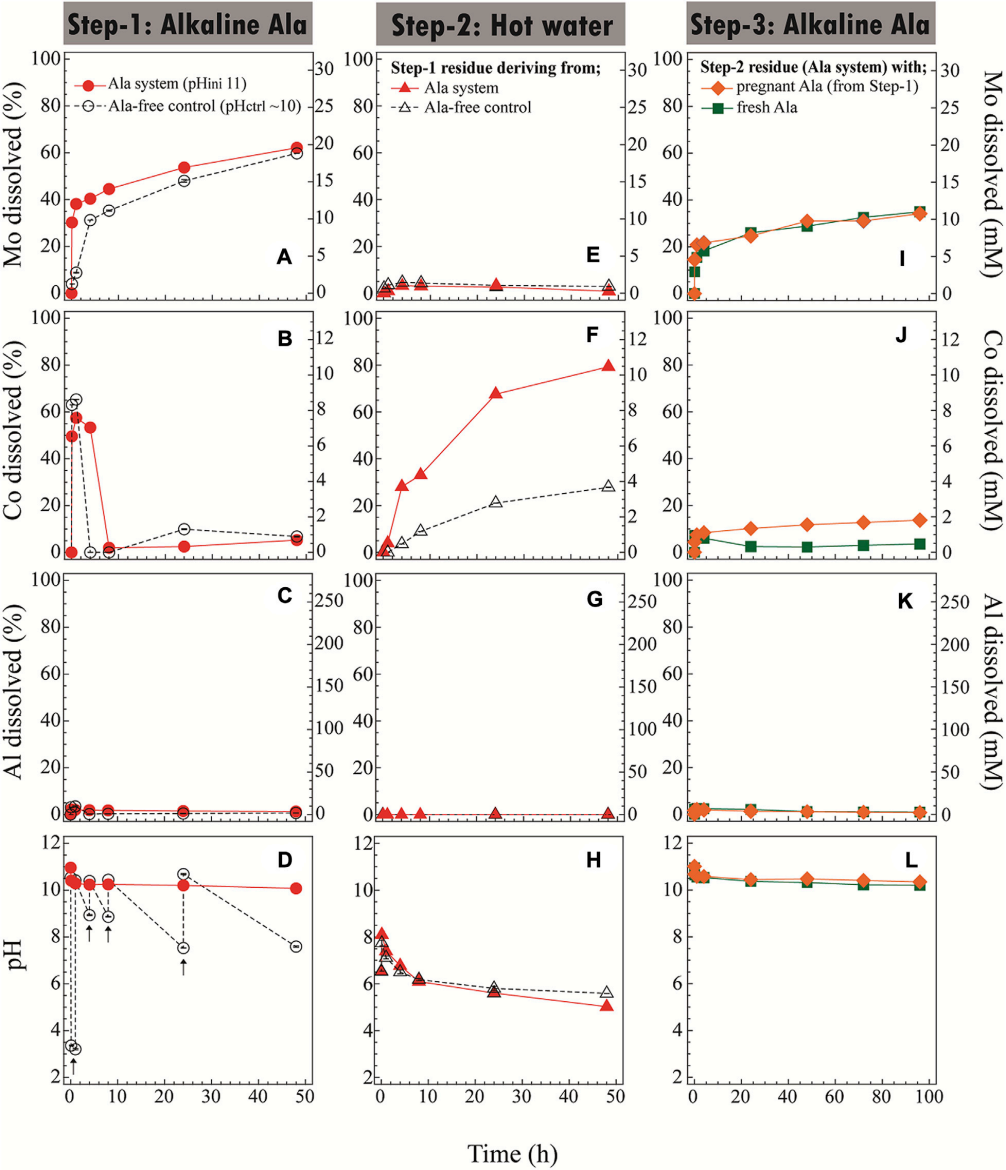
Sequential 3-step leaching of spent catalyst. Step-1 **(A–D)**: Alkaline Ala leaching (pHini 11; ●) or Ala-free control [pH adjusted to ∼10 with NaOH, as indicated by arrows **(D)**; ○] at 45°C. Step-2 **(E–H)**: Hot water leaching of the Step-1 solid residue [deriving from Ala system (▲) or Ala-free control (△)] at 70°C, pHnatutal. Step-3 **(I–L)**: Second alkaline Ala leaching of the Step-2 solid residue (deriving from Ala system in Step-1), either by reusing pregnant Ala solution from Step-1 (re-adjusted to pHini 11; 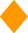) or by using fresh Ala solution (pHini 11; 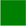) at 45°C. The dissolution profile of Mo **(A,E,I)**, Co **(B,F,J)**, and Al **(C,G,K)** as well as the pH profile **(D,H,L)** are shown.

As described in the previous section, Ala was found to be a possible candidate to selectively leach and recover Mo and Co while suppressing the Al dissolution. To clarify the role of Ala, the control test using Ala-free alkaline water (pH_ini_ 11) was run in parallel. In the Ala-free system, pH_ini_ 11 rapidly dropped to acidic, so it was necessary to add alkaline manually (at 1, 4, 8, and 24 h) to keep the equivalent pH level to the Ala system (Figure 3D). It was reported that Mo dissolves from MoS_2_ in alkaline solution following the Eqs 3, 4 as below (Mirvaliev and Inoue, 2001), thus acidifying the leachate. However, after pH_ini_ 11 dropped slightly to ∼10, no further pH adjustment was necessary for the Ala system (Figure 3D) since the pH level was stabilized after that due to the buffering effect of amino acid molecules.

$${MoS}_{2}+{3H}_{2}O+{4.5O}_{2}={HMoO}_{4}^{-}+{2SO}_{4}^{2-}+{5H}^{+}$$ (3)

$${HMoO}_{4}^{-}+{5H}^{+}+{6OH}^{-}={MoO}_{4}^{2-}+{6H}_{2}O$$ (4)

Although the final Mo dissolution was nearly equal (∼60% at 48 h), the initial leaching effect was quicker and continued to be more stable in the presence of Ala compared to the Ala-free control (Figure 3A). This may be due to the stable pH level maintained by Ala, as well as a possible complexolysis effect of Ala with Mo. The leaching mechanism involving the complexation of metals with the simplest amino acid, Gly, was reported (Eksteen et al., 2017; Li et al., 2020). Djordjevic et al. (1997) chemically synthesized the Mo(IV) complex with various amino acids, including Mo-Ala. However, whether or not the formation of the Mo-Ala complex occurs during the leaching reaction (such as in this study) remains to be clarified in further investigations.

**FIGURE 4:**
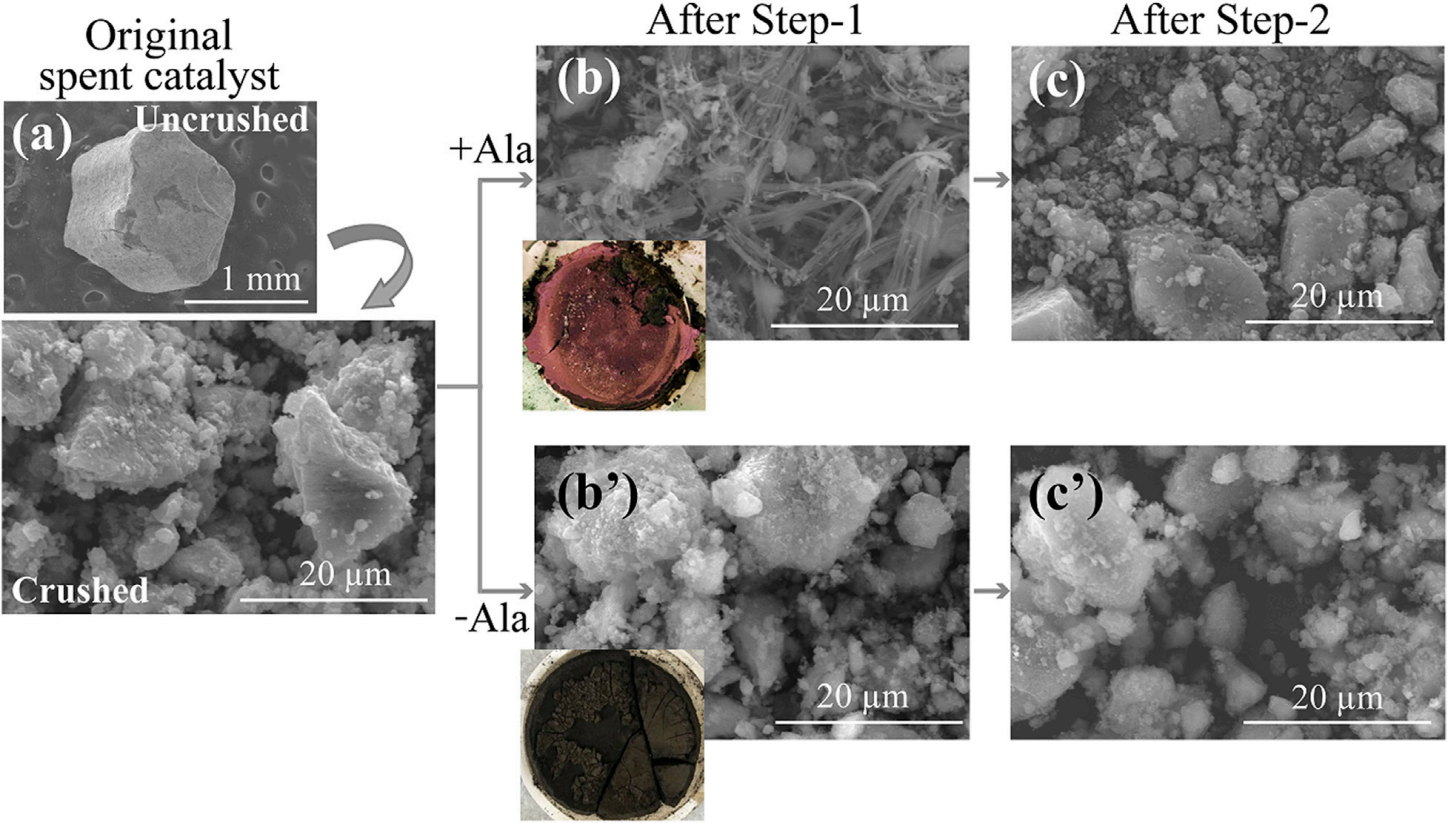
SEM images showing morphological and colorimetric differences of Co-precipitates formed during the Step-1 and their transition during Step-2. **(A)** Original spent catalyst (before and after crushing). **(B,B′)** The leaching residue after Step-1 **(B)**; Ala system, **(B′)**; Ala-free control. **(C,C′)** The leaching residue after Step-2 **(C)**; Ala system applied in Step-1, **(C′)**; Ala-free control system applied in Step-1.

In both conditions (with or without Ala), about 60% of Co was found to solubilize during the first few hours and then continued to precipitate thereafter. At 48 h, only 5% of Co was soluble under both conditions (Figure 3B). From the acidic and weak alkaline solution, Co tends to exist as Co^2+^ but precipitate as Co-hydroxides at elevated pHs (Huang et al., 2004). Without Ala, therefore, Co was likely precipitated as Co-hydroxides of blackish brown color (Figure 4B′), which was not easily distinguishable from the spent catalyst particles. On the other hand, the presence of Ala altered the nature of precipitate to exhibit the distinctive pink color and characteristic fiber-like structure (Figure 4B). The formation of secondary Co-precipitate was also evidenced by the emergence of new, unknown crystalline XRD peaks after Step-1 (Figure 1B). Since the acidity constant of Ala is pKa_2_ = 9.69, its carboxylic functional group tended to deprotonate at the reaction pH of ∼10 in this test, leading to the complexation of Co^2+^ with two negatively charged Ala molecules and precipitate (Alam et al., 2019).

The overall dissolution of Al was low throughout the leaching period in Step-1; only 1.3% or 0.7% Al was found to be soluble with or without Ala, respectively, at 48 h (Figure 3C). The pH level seemed to play a major role in the Al dissolution as the presence of Ala only had a marginal effect. In an alkaline solution, Al_2_O_3_ was reported to dissolve at pH 10.5 as Al(OH)_4_^−^ (Pinto and Soares, 2012). A small portion of Al (∼3%) was dissolved at the starting point in Step-1 but then seemingly precipitated as AlO(OH), as was confirmed by XRD (Figures 1B–D). Overall, in Step-1, the dissolution trends of Mo, Co and Al were seemingly similar regardless of the presence of Ala. However, the buffering capacity of Ala effectively maintained the alkalinity in the leachate without necessitating a continuous pH re-adjustment.

Applying different temperatures (25, 45 or 70°C) to Step-1 affected the Mo dissolution and, more significantly, the Co-precipitation behavior (Supplementary Figures S4A,B). Lower temperature (25°C) seemed to stabilize Co-precipitate while higher temperature (70°C) inhibited Co-Ala complexation. O’Connor et al. (2018) and Li et al. (2020) also reported the decomposition of aqueous Gly, the simplest amino acid, at temperatures such as 55 and 60°C. In this case, applying a moderate temperature (45°C) was shown to be most effective in terms of Mo dissolution and Co-precipitation (Supplementary Figure S4).

##### 3.3.1.2 Step-2: Hot water leaching

This step attempted to compare the leachability of Co from the Step-1 residue (mixture of spent catalyst plus Co-precipitate formed with or without Ala) by hot water treatment (pH natural; 70°C). The leachability of Co from Ala-free Co-precipitate was significantly lower (28%) than that from Co-Ala precipitate (79%) (Figure 3F). The greater Co leachability from the latter may have resulted from the decomposition of Ala (through deamination and decarboxylation to produce mainly lactic acid and ethylamine; Klingler et al., 2007) in hot water, leading to the release of free Co^2+^ in the leachate. The dissolution of Mo and Al from the spent catalyst was negligible in Step-2 (Figures 3E,G). In fact, applying lower temperatures (25 or 45°C) resulted in increasingly lower Co^2+^ release (Supplementary Figure S5B), suggesting that higher temperature is favorable to resolubilize Co-Ala precipitates. A decrease in pH was seen during Step-2 regardless of the presence of Ala (Figure 3H). This was possibly caused by the slight dissolution of spent catalyst particles.

The decomposition of Co-Ala precipitates was also suggested by XRD (Figure 1C) and SEM (Figure 4C): XRD peaks emerged during Step-1 (deriving from Co-Ala precipitates; Figure 1B) disappeared during Step-2 (Figure 1C), accompanied by the disappearance of distinctive fiber-like structures (Figure 4C). In contrast, most of the amorphous Co-precipitates formed in the Ala-free control (Figure 1B'; Figure 4B') persisted the hot water treatment in Step-2 (Figure 4C'). This was consistent with the lower Co dissolution in the Ala-free system as shown in Figure 3F. Overall, the advantage of using the Ala system in Step-1 was emphasized in Step-2 in that Co^2+^ can be selectively released by a simple water treatment through the resolubilization of Co-Ala precipitates.

##### 3.3.1.3 Step-3: Second alkaline L-alanine leaching

This final step attempted to leach out remaining Mo (∼37%) from the Step-2 residue by applying the alkaline Ala solution for the second time. Two types of Ala solution (one reusing pregnant Ala solution from Step-1, the other using fresh Ala solution) were compared. The Mo dissolution progressed nearly to completion in 96 h, while the Al dissolution was largely suppressed in both reused and fresh Ala solutions (Figures 3I,K). However, the Co dissolution trend was different between the two; i.e., the remaining Co in the spent catalyst dissolved and precipitated in the fresh Ala solution (as was seen in Step-1), but Co did not precipitate in the pregnant Ala solution (Figure 3J). This may have been caused by the less availability of free Ala molecules (to be precipitated with Co) in the pregnant Ala solution because Ala could have been consumed in Step-1 not only by Co but also by Al and/or Mo through complexation. It can be speculated that the number of free Ala molecules was stoichiometrically enough for the chelation with Co, but not enough to thermodynamically stabilize the complex for precipitation. Another factor could be the effect of temperature (45°C, in this case) after a certain period of incubation on the stability of Ala molecules. To clarify this, further studies are ongoing to investigate the stability and reusability of amino acid lixiviant.

#### 3.3.2 Chemical synthesis and comparison of Co-Ala complexes

**FIGURE 5:**
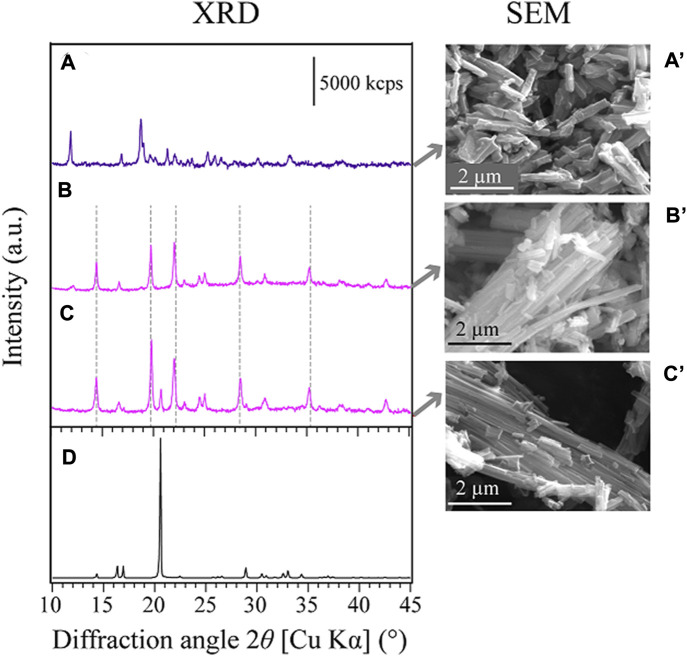
Comparison of XRD patterns **(A–D)** and SEM images **(A′–C′)** of synthesized Co-Ala precipitates: **(A,A′)** Co-Ala complex produced by the ethanol reflux method at 80°C. **(B,B′)** Co-Ala complex produced from pure Ala and Co^2+^ reagents at 45°C. **(C,C′)** Co-Ala complex formed in the actual spent catalyst leachate (spent catalyst particles separated prior to Co-Ala precipitation) at 45°C. **(D)** Pure Ala reagent. **(A–C)** All XRD peaks derived from the synthesized Co-Ala complexes under each condition and did not match the database.

As described in Section 3.3.1.1, the XRD peaks of the Co-precipitate formed in Step-1 did not match the XRD database (Figure 1B). Hence, a separate test was done to obtain chemically-synthesized Co-Ala complexes as reference materials to confirm its identity. XRD peaks (Figures 5A–C) and SEM images (Figures 5A′–C′) of three different Co-Ala preparations are shown together with XRD peaks of pure Ala reagent (Figure 5D).

**FIGURE 6:**
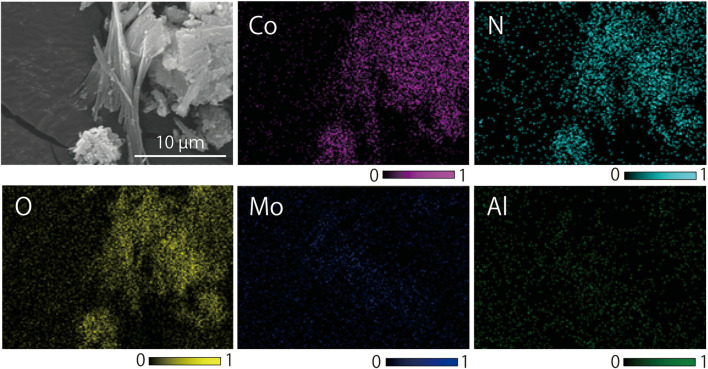
SEM-EDS elemental mapping of Co-Ala complex formed in the actual spent catalyst leachate (spent catalyst particles separated prior to Co-Ala precipitation) at 45°C (corresponding to Figures 5C,C′): The secondary electron image was mapped for Co, N, O, Mo and Al.

The Co-Ala sample obtained from the ethanol reflux method at 80°C (Alam et al., 2019; modified) exhibited a purple color with shorter needles (Figure 5A′). Its XRD peak positions (Figure 5A) differed from those obtained under milder conditions (45°C; Figures 5B,C). The Co-precipitate formed in the particle-free spent catalyst leachate (Figures 5C,C′) displayed nearly identical color (pink), morphology (long needle-like crystals), and XRD peaks to that formed from pure Co and Ala reagents (Figures 5B,B′) under similar physicochemical conditions (temperature, concentrations). This suggests that the Co-precipitate formed during Step-1 (Section 3.3.1.1) indeed consisted of Co and Ala. Additionally, Figure 6 reveals the elemental composition of the Co-precipitate formed in the spent catalyst leachate: Co, as well as N and O (originating from amino acid molecules), were shown to be the component of this precipitate. The presence of other metals such as Mo and Al was negligible.

#### 3.3.3 Effect of different sequential order

Before selecting the above 3-step order (Section 3.3.1), preliminary tests were conducted to compare different sequential orders. Supplementary Figure S6 shows the results from a different 3-step order (Step-1: Alkaline Ala leaching, Step-2: Second alkaline Ala leaching, Step-3: Hot water leaching). The leaching trend of Mo in alkaline Ala solutions was relatively similar between the two different sequential orders (Supplementary Figures S6A,E; Figures 3A,I). However, applying the hot water treatment as Step-3 (Supplementary Figure S6J) instead of Step-2 (Figure 3F) significantly lowered the solubilization of Co^2+^ from Co-Ala precipitates. This was likely caused by a pH spike during the hot water leaching in Step-3 (Supplementary Figure S3L), owing to an excessive alkalization of spent catalyst particles in Step-1 and Step-2. Setting the hot water Co leaching at Step-1 was also found to be unfavorable since Co dissolution was accompanied by co-dissolution of a certain level of Mo and Al (data not shown). Overall, the sequential leaching order described in Section 3.3.1 was found to be most effective in terms of the total metal dissolution as well as metal selectivity.

#### 3.3.4 Process overview and comparison with previous studies

**FIGURE 7:**
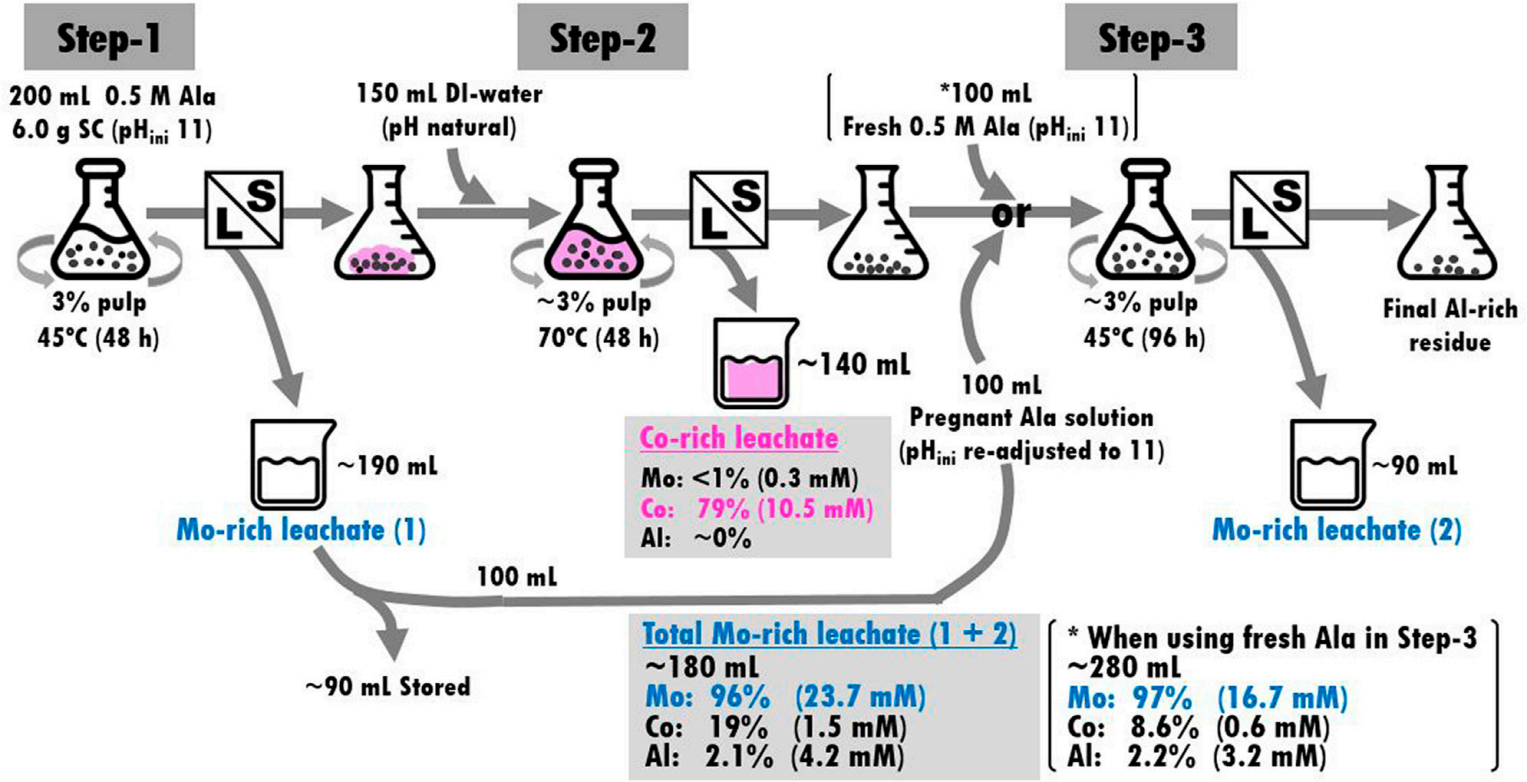
The overview of 3-step leaching flow and the resultant metal contents of Co-rich and Mo-rich leachates.

Figure 7 summarizes the process flow overview of the sequential 3-step leaching described in Section 3.3.1. Two separate leachates, “Co-rich leachate” and “Total Mo-rich leachate” were eventually obtained with highly selective metal dissolutions (Figure 7). Reusing the pregnant Ala solution in Step-3 was favorable in terms of more concentrated Mo content and cost-effectiveness, although the metal selectivity between Mo and Co became slightly lowered (Figure 7).

When calculated on the liquid analysis basis (Figures 3, 7), the whole 3-step leaching process solubilized a total of ∼97% Mo, 98% Co and ∼2.1% Al from the original spent catalyst (*E*_*l*_; Table 2). In order to verify these values, the final Al-rich Step-3 residue was acid digested, followed by ICP-OES analyzed to calculate its metal mass (Table 2). This solid-based calculation led to the total metal dissolution of 90% Mo, 93% Co and 1.5% (Table 2). Although a slight overestimation was found when calculated solely on the liquid analysis basis, the overall tendency was consistent.

**TABLE 2:** Total metal dissolution calculated based solely on the liquid analysis or the combination of liquid and solid analyses.

	Original spent catalyst	Final Al-rich residue (after Step-3 reusing pregnant Ala solution)		
	Metal mass^a^ (*m_o_*, mg/g)	Metal mass^a^ (*m_r_*, mg/g)	Total metal dissolution (Sum of Step-1, 2, and 3);	
			Caluculated from liquid analysis (Figure 7) (*E_l_*, %)	Calculated from liquid + solid analysis (*E_m_*, %)
Mo	101 ± 8	10.8 ± 0.02	97	90
Co	24 ± 0.8	1.7 ± 0.03	98	93
Al	248 ± 2.7	359 ± 0.6	2.1	1.5

$E_{m}=m_{f}/\left(m_{f}+m_{r}\right)\times$ 100% Li et al. (2020). $m_{f}=E_{l}\%m_{o}$ (the mass of dissolved metal, calculated based on the liquid analysis).

a Solid analysis data (acid digestion followed by ICP-OES analysis).

Overall, the role of alkaline Ala solution in the process was three-fold; 1) maintaining alkalinity in Step-1 and Step-3 by amino acid’s buffering capacity to assist Mo leaching, 2) selectively precipitating Co by forming Co-Ala complex, which can readily re-dissolve in hot water to be separated from spent catalyst particles. 3) suppressing dissolution of Al throughout the reaction without needing pH control. In addition, the possibility of the Mo-Ala complexation effect cannot be exempted at this stage. Further studies are necessary to clarify this.

So far, several literatures have reported the use of inorganic and organic lixiviant for the dissolution of critical metals (Mo, Co, Ni) from spent hydrodesulfurization catalysts (Table 3). Angelidis et al. (1995) attempted to selectively recover Mo, Co, and Ni using a two-step alkali-acid leaching. Mo and Co were selectively dissolved (96% and 91%, respectively), but the Al dissolution was not well controlled (Table 3). Barik et al. (2012) attempted to leach Mo and Co from spent catalyst using H_2_SO_4_ by adding various oxidants. When using H_2_O_2_ as an oxidant, Mo and Co were nearly completely but non-selectively leached (99.8% and 96.2%, respectively), accompanied by 11% Al dissolution (Table 3). Pinto and Soares (2012) applied NaOH-based microwave treatment for four cycles, resulting in the dissolution of 91% Mo and 8.8% Al. Pinto and Soares (2013) then used organic chelating agents (Ethylenediaminetetraacetic acid, EDTA; Nitrilotriacetic acid, NTA) under the microwave condition to selectively leach Ni from Ni-Mo spent catalyst. EDTA gave a better Ni dissolution (80%) than NTA (64%), with some co-solubilization of Mo and Al (Table 3). Arslanoǧlu and Yaraş (2019) applied formic acid for the roasted spent Mo-Co-Ni/Al_2_O_3_ catalyst, wherein 76% Mo, 97% Co, and 94% Ni were leached with relatively high co-solubilization of Al (19%) (Table 3). Compared to the previous reports summarized in Table 3, the results obtained in this study achieved highly selective leaching of critical metals from spent desulfurization catalyst.

**TABLE 3:** Comparison of the spent hydrodesulfurization catalyst leaching studies.

Spent catalyst	Lixiviant		Condition		Metal dissolution, %			References
	Type	Conc.			Mo	Co/Ni	Al	
Mo-Co/Al_2_O_3_	NaOH, H_2_SO_4_	10 g/L	Two-step alkali and acid leaching	Alkali leachate	96*	0.09/‐	14	Angelidis et al. (1995)
				Acidic leachate	0.03	91^a^/‐	54	
Mo-Co/Al_2_O_3_	H_2_SO_4_	0.5 mol/L	Leaching reactor		99^a^	96^a^/‐	11	Barik et al. (2012)
Mo-Co/Al_2_O_3_	NaOH	10 g/L	Microwave assisted		91^a^	N.D.	8.8	Pinto and Soares (2012)
Mo-Ni/Al_2_O_3_	EDTA	0.1 mol/L	Microwave assisted		10	‐/80^a^	2.8	Pinto and Soares (2013)
Mo-Ni/Al_2_O_3_	NTA	0.2 mol/L	Microwave assisted		8	‐/64^a^	1.8	Pinto and Soares (2013)
Mo-Co-Ni/Al_2_O_3_	Formic acid	0.6 mol/L	Roasting and leaching		76^a^	97/94^a^	19	Arslanoğlu and Yaraş (2019)
Mo-Co/Al_2_O_3_	Alanine, H_2_O	0.5 mol/L	Sequential leaching	Co-rich leachate	<1	79^a^/‐	0	This study
				Total Mo-rich leachate	96^a^ ^, P^ (97^F^)	19^P^ (8.6^F^)/‐	2.1^P^ (2.2^F^)	

a Target metal. P, Pregnant Ala solution reused in Step-3. F, Fresh Ala solution used in Step-3. N.D., No data available. -,.Not applicable.

## 4 Conclusion

The spent hydrodesulfurization catalyst sample used in this study consisted of MoS_2_ and Co_3_S_4_ supported on Al_2_O_3_, at the Mo and Co grade of 10% and 2.4%, respectively. In contrast to the case of H_2_SO_4_ and citric acid, alkaline amino acids generally suppressed the dissolution of unwanted Al. Still, the leaching trend of Mo and Co differed largely depending on the type of amino acids.

Alanine was the most effective, simple amino acid in supporting the Mo alkalosis by its buffering effect while simultaneously precipitating Co as Co-Ala complex of distinctive color and morphology, enabling selective recovery of Mo-rich leachate (96% Mo, 19% Co, and 2.1% Al dissolved). The formation of the Co-Ala complex was favorable in terms of its solubility in hot water (supposedly due to the decomposition of amino acids), enabling selective recovery of Co-rich leachate (<1% Mo and 79% Co dissolved, Al not detected). Consequently, the sequential 3-step process (Step-1: Alkaline Ala leaching at 45°C, Step-2: Hot water leaching at 70°C, Step-3: Alkaline Ala leaching at 45°C) achieved highly selective leaching of critical metals (Mo and Co) compared to previous studies.

## Data Availability

The original contributions presented in the study are included in the article/Supplementary Material, further inquiries can be directed to the corresponding author.

## Supplementary material

SUPPLEMENTARY FIGURE S1 Pourbaix diagram for Molybdenum—H_2_O system. ∑ Mo = 10^−5^ M at 25°C (Lyon, 2010).

SUPPLEMENTARY FIGURE S2 Pourbaix diagram for Cobalt—H_2_O system. ∑ Co = 10^−6^ M at 25°C (Chivot et al., 2008).

SUPPLEMENTARY FIGURE S3 Pourbaix diagram for Aluminum—H_2_O system. ∑ Al = 3 × 10^−6^ M at 25°C (Pourbaix, 1974; Sukiman et al., 2012).

SUPPLEMENTARY FIGURE S4 The effect of different temperatures (25, 45, and 70°C) on Step-1.

SUPPLEMENTARY FIGURE S5 The effect of different temperatures (25, 45, and 70°C) on Step-2.

SUPPLEMENTARY FIGURE S6 Sequential 3-step leaching of spent catalyst (Step-2 and Step-3 reversed from Figure 3). Step-1 **(A‐D)**: Alkaline Ala leaching (pH_ini_ 11) at 45°C. Step-2 **(E–H)**: Second alkaline Ala leaching of the Step-1 solid residue in fresh Ala solution (pH_ini_ 11) at 45°C. Step-3 **(I–L)**: Hot water leaching of the Step-2 solid residue at 70°C. The dissolution profile of Mo **(A,E,I)**, Co **(B,F,J)**, and Al **(C,G,K)** as well as the pH profile **(D,H,L)** are shown.
