# Supplementary figures and images for: Utilization of amino acid for selective leaching of critical metals from spent hydrodesulfurization catalyst
Source: Front Chem. 2022 Oct 10;10:1011518. doi: 10.3389/fchem.2022.1011518 (PMC9592186; doi:10.3389/fchem.2022.1011518)

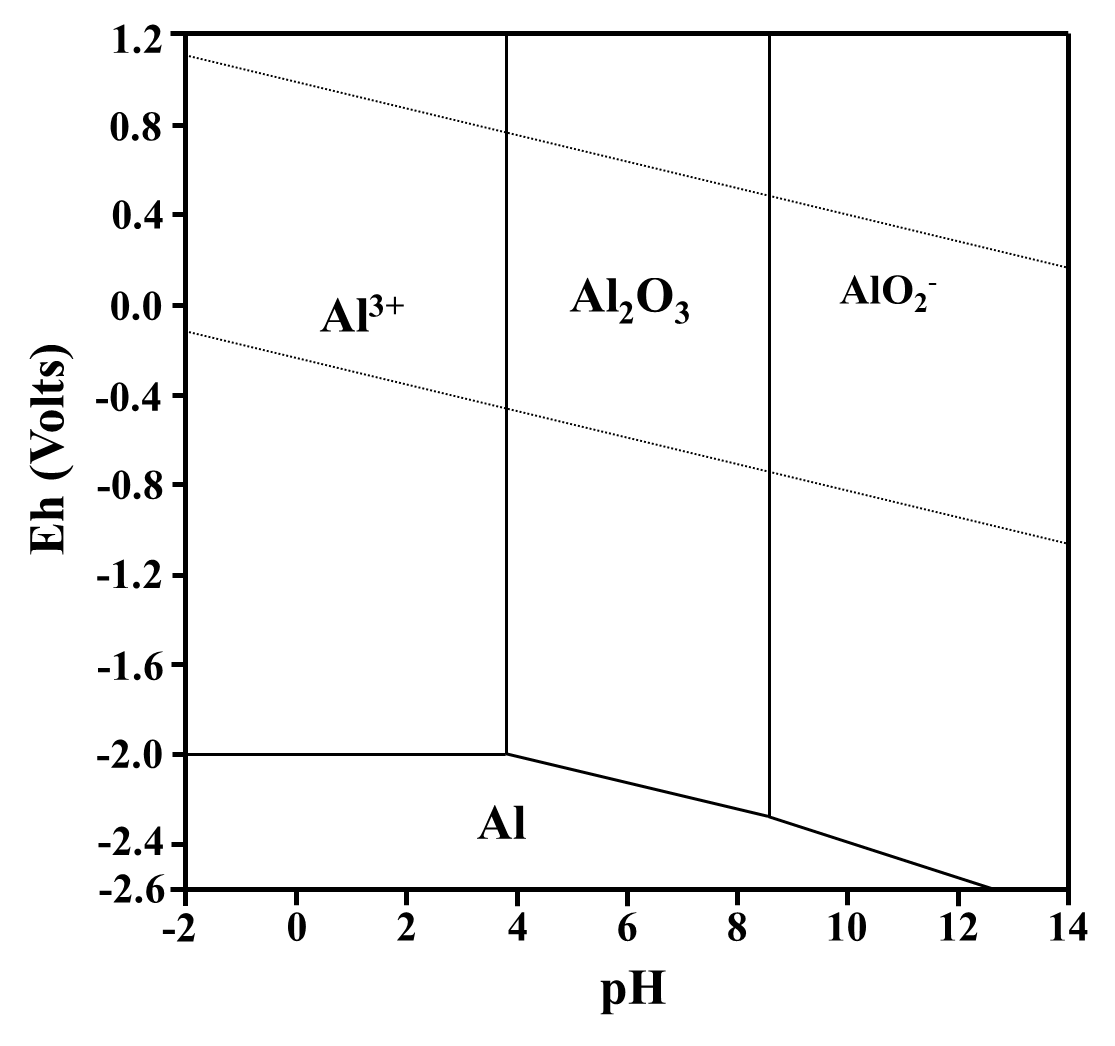

Supplement: Supplementary file 1 [file Image3.tif]

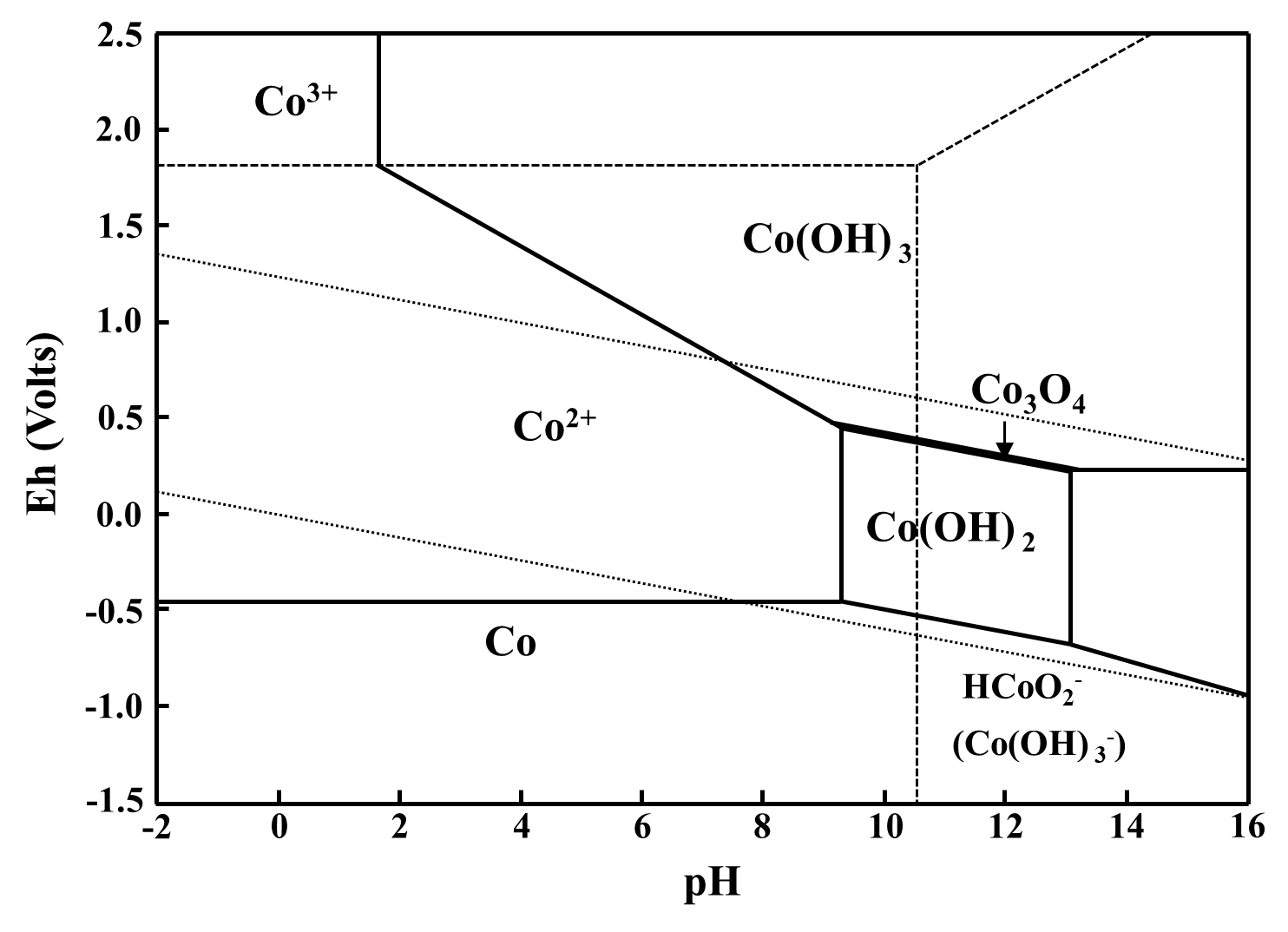

Supplement: Supplementary file 3 [file Image2.tif]

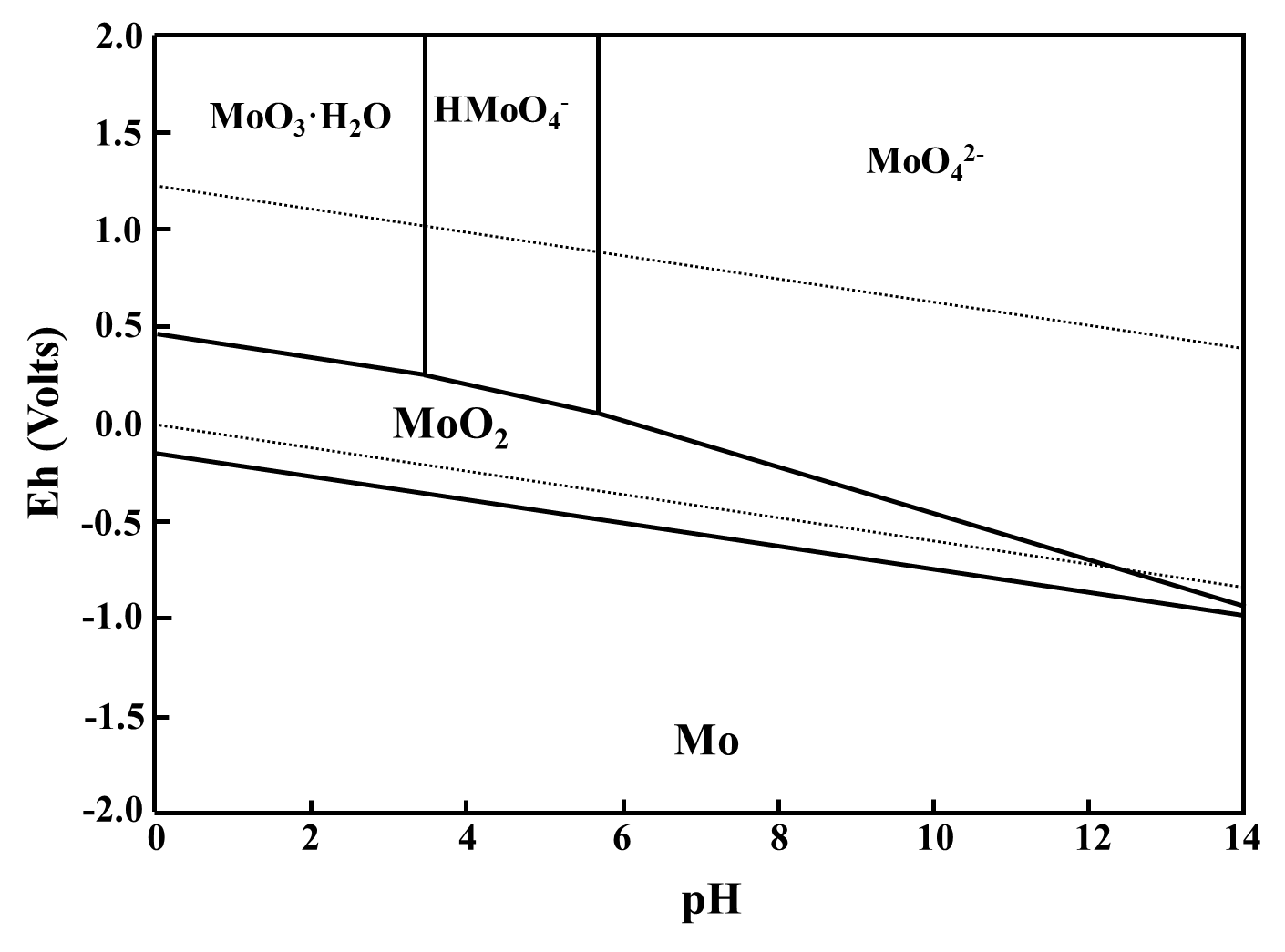

Supplement: Supplementary file 4 [file Image1.tif]
